# Phylogeographic Insights into *Aedes albopictus* in Korea: Integrating COX1, ND5, and CYTB Analyses

**DOI:** 10.3390/insects17010082

**Published:** 2026-01-10

**Authors:** Sezim Monoldorova, Jong-Uk Jeong, Sungkyeong Lee, Ilia Titov, In-Yong Lee, Hojong Jun, Jin-Hee Han, Fauzi Muh, Kwang-Jun Lee, Bo-Young Jeon

**Affiliations:** 1 Department of Biomedical Laboratory Science, College of Software and Digital Healthcare Convergence, Yonsei University, Wonju 26493, Republic of Korea; sezima91@gmail.com (S.M.);; 2 One Health Frontiers, Co., Ltd., Wonju 26493, Republic of Korea; 3 Department of Medical Environmental Biology and Tropical Medicine, School of Medicine, Kangwon National University, Chuncheon 24341, Republic of Korea; 4 Department of Epidemiology and Tropical Diseases, Faculty of Public Health, Universitas Diponegoro, Semarang 50275, Indonesia; 5 Division of Zoonotic and Vector Borne Disease Research, Center for Infectious Diseases Research, Korea National Institute of Health, Cheongju 28159, Republic of Korea

**Keywords:** *Aedes albopictus*, cytochrome *c* oxidase subunit 1 (COX1), NADH dehydrogenase subunit 5 (ND5), cytochrome *b* (CYTB), phylogenetic diversity

## Abstract

The Asian tiger mosquito (*Aedes albopictus*) is an important vector of arboviruses, including dengue, chikungunya, and Zika. Its rapid global expansion has been facilitated by climate change and human activities. Phylogenetic studies of *Ae. albopictus* have largely relied on mitochondrial cytochrome *c* oxidase subunit 1 (COX1) and NADH dehydrogenase subunit 5 (ND5) markers, while the utility of cytochrome *b* (CYTB) remains underexplored. We collected *Ae. albopictus* from 13 sites in seven provinces of South Korea and analyzed COX1, ND5, and CYTB sequences. Genetic diversity indices were calculated, and phylogenetic relationships were reconstructed using maximum-likelihood trees and haplotype networks with a dataset obtained from GenBank. COX1 revealed 46 haplotypes, including six novel variants, with the highest diversity in southern coastal regions such as Busan and Suncheon. ND5 showed limited variation, with only two haplotypes. CYTB revealed three haplotypes, including region-specific variants in Busan and Wonju, supporting its role as a complementary marker. The Busan haplotype H41 bridged domestic and international lineages, suggesting Busan as a likely entry point. This study demonstrates that integrating COX1, ND5, and CYTB improves the resolution of *Ae. albopictus* phylogeography in Korea and highlights the need for continued molecular surveillance to guide vector control strategies.

## Simple Summary

The Asian tiger mosquito (*Aedes albopictus*) is an invasive species that can spread medically important arboviruses such as dengue, chikungunya, and Zika. Its ability to expand worldwide is strongly influenced by climate change and human travel. In South Korea, this mosquito is widely distributed, but few studies have integrated multiple mitochondrial markers. In this study, we collected mosquitoes from 13 locations in seven provinces and analyzed their genetic material using three markers: COX1, ND5, and CYTB, which are useful for tracing the relationship among mosquito populations and understanding how they may spread between regions. Our results showed high genetic diversity in southern coastal areas such as Busan and Suncheon, suggesting these regions may serve as entry points for new introductions. The findings also revealed distinct groups in mainland Korea and Jeju Island. Using multiple genetic markers provided an understanding of mosquito diversity and their links to international populations, supporting mosquito surveillance and effective control strategies for *Ae. albopictus* in Korea and beyond.

## 1. Introduction

Vector-borne diseases transmitted by arthropods such as mosquitoes, ticks, and sandflies remain a major public health concern. Climate change—particularly increases in global temperatures, altered rainfall patterns, and rising humidity—affects their transmission by enhancing the survival, reproduction, and geographic expansion of vectors [1,2]. Dengue fever is endemic in tropical and subtropical regions, and its spread is driven by climate change, rapid urbanization, and increasing population density [3]. A notable dengue outbreak in Japan in 2014, despite having no prior history of dengue outbreaks [4]. Chikungunya, originally endemic to Africa, was first reported outside the continent during an outbreak in Italy in 2007 [5], and has since spread across tropical and subtropical regions in Europe, Asia, and South America, causing multiple epidemics with increasing frequency [6]. In Korea, malaria and Japanese encephalitis remain the primary mosquito-borne diseases of concern [7]. Imported cases of dengue, chikungunya, and Zika virus infections continue to be reported [8], raising concern about the potential introduction and establishment of these diseases [9].

Major vectors capable of transmitting arboviruses such as dengue, Zika, and chikungunya include Yellow fever mosquito (*Aedes aegypti*, *Ae*. *aegypti*) and the Asian tiger mosquito (*Ae. albopictus*). The *Ae*. *aegypti* mosquito is highly anthropophilic, breeding primarily in artificial containers around human dwellings, and is a major vector of dengue fever, Zika virus, chikungunya virus, and yellow fever [8]. *Ae*. *aegypti* is widely distributed across tropical and subtropical regions, including Africa, the Americas, South and Southeast Asia, and the Pacific islands. However, its distribution is significantly constrained by temperature and humidity because it relies heavily on human habitats and relatively warm environments [10].

By contrast, the Asian tiger mosquito (*Ae. albopictus*), native to Asian forests, is more ecologically versatile [11]. It can utilize both natural and artificial containers, tolerate a broader range of climates, and establish itself in peri-urban and rural environments. Like *Ae*. *aegypti*, it is a competent vector of multiple arboviruses, but it is considered far more invasive [12]. Since the 1980s, *Ae. albopictus* has expanded rapidly into the Americas, Europe, and many Pacific islands. The ability of temperate *Ae. albopictus* strains to survive freezing temperatures and overwinter as diapausing eggs has facilitated its establishment in cooler temperate regions where *Ae*. *aegypti* cannot persist [13]. The continued global spread of *Ae. albopictus* underscores the growing potential for arboviral transmission at higher latitudes [2,14].

Determining the routes of mosquito introduction is critical for understanding the global spread of invasive vector species. Phylogenetic analysis has been employed to investigate the population structure of mosquitoes, identify distinct haplotypes, and elucidate both regional differentiation and international genetic relationships. For this purpose, mitochondrial DNA has been widely employed in phylogenetic studies owing to its maternal inheritance and relatively rapid mutation rate [15]. The mitochondrial genome of *Ae. albopictus* is 16,665 bp in length, and the COX1 (cytochrome *c* oxidase subunit 1) gene is among the most frequently used markers for phylogenetic analyses, owing to its standard barcode region, high mitochondrial mutation rate, and strong discriminatory power [16]. Phylogenetic analyses using the COX1 gene have provided evidence for the species’ recent global expansion [17]. NADH dehydrogenase subunit 5 (ND5) has been reported as a useful marker for population and geographical phylogenetic analyses of *Ae. albopictus* in Malaysia [18] as well as in Cameroon, Hawaii, Brazil, and the United States [19]. However, a study by Palacio-Cortés in Brazil reported a relatively low ND5 genetic diversity [20]. Cytochrome *b* (CYTB) is a useful phylogenetic marker across insects, including mosquitoes [21], and has been used to analyze population structure and patterns in other mosquito genera, including *Anopheles* and *Culex* [22]. In *Ae. albopictus*, a phylogenetic study of Mexican populations using CYTB revealed substantial genetic variation and provided insights into invasion pathways [23], whereas other studies have reported relatively low CYTB diversity in this species [18]. 

In Korea, phylogeographic analyses of *Ae. albopictus* have primarily relied on COX1 sequences [24,25]. Although recent studies have incorporated both COX1 and ND5, the phylogenetic resolution of ND5 remains unclear [25], and no studies have evaluated CYTB in Korean *Ae. albopictus* populations. Phylogenetic investigations of the introduction routes of *Ae. albopictus* population in Korea are still limited.

Therefore, in this study, we assessed the phylogeographic utility of CYTB—together with ND5 and COX1—for *Ae. albopictus* populations in Korea and used these markers to investigate the geographic invasion into Korea.

## 2. Materials and Methods

### 2.1. Mosquito Collection and Identification

To investigate the genetic diversity of Asian tiger mosquitoes (*Ae. albopictus*) in the Republic of Korea, adult mosquitoes were collected from 13 sites across seven provinces. A portion of the specimens was obtained through the mosquito-borne disease surveillance program conducted by the Korea Centers for Disease Control and Prevention (KDCA). Mosquitoes were collected from 15:00 to 10:00 (overnight) using BG-Sentinel™ traps (Biogents AG, Regensburg, Germany) baited with BG-Lure^®^ and carbon dioxide supplied via dry ice. 

The collection sites were as follows: Chuncheon, Donghae, Gangneung, Samcheok, Sokcho, and Wonju in Gangwon Province; Dangjin, and Geumsan in Chungnam Province; Chungju in Chungbuk Province; Suncheon in Jeonnam Province; Jinju in Gyeongnam Province; and Goryeong in Gyeongbuk Province, and Busan Metropolitan City (Table S1).

A total of 4658 adult mosquitoes were collected and morphologically identified to species level by light microscopy using the taxonomic key described by Ree [26] (Table S1). Of the 542 *Ae. albopictus* collected, 54 individuals were selected for genetic analyses to ensure high-quality DNA and balanced regional representation.

### 2.2. Ethical Approval

All procedures involving the collection and handling of mosquitoes were reviewed and approved by the Institutional Animal Care and Use Committee (IACUC) of Yonsei University Mirae Campus (Approval No. YWCI-202005-007-02). All experiments were conducted in accordance with the IACUC guidelines.

### 2.3. DNA Extraction, PCR Amplification, and Sequencing

Mosquitoes morphologically identified as *Ae. albopictus* were washed twice in 70% ethanol and placed into reinforced homogenizing tubes (Bertin, Montigny-le-Bretonneux, France) containing 2.8 mm zirconium oxide beads (Bertin) and 200 μL of TRIzol reagent (Invitrogen, Waltham, MA, USA). Mosquitoes were chilled at 4 °C for 30 min prior to homogenization, and homogenized twice at 5000 rpm for 20 s using a mechanical homogenizer (Precellys Evolution Touch Homogenizerl, Bertin Technologies, Montigny-le-Bretonneux, France). Genomic DNA was extracted using the G-spin™ Total DNA Extraction Mini Kit (Intron Biotechnology Inc., Seongnam, Republic of Korea) according to the manufacturer’s instructions and stored at −80 °C until further use.

To analyze the genetic diversity of *Ae. albopictus*, variable regions of three mitochondrial genes—COX1, ND5, and CYTB—were targeted [27,28]. Primers newly designed using Primer3 software (https://bioinfo.ut.ee/primer3-0.4.0 (accessed on 5 May 2020)) were used to amplify COX1 (nucleotide positions 2219–2966) and the CYTB (10,426–10,963) of the GenBank mitochondrial genome of *Ae. albopictus* (GenBank accession no. NC_006817) (Table S1).

PCR amplification was performed in a total volume of 20 μL using the AccuPower^®^ PCR PreMix kit (Bioneer Co., Daejeon, Republic of Korea), which contains Taq DNA polymerase and dNTPs, with 2 μL of each primer (10 pmol/μL), 2 μL of template DNA, and 14 μL of distilled water. PCR amplification conditions for COX1 and ND5 consisted of an initial denaturation at 95°C for 5 min, followed by 35 cycles of 94 °C for 30 s, 55 °C for 60 s, and 72 °C for 60 s, with a final extension at 72 °C for 7 min. For CYTB, amplification was performed with an initial denaturation at 95 °C for 10 min, followed by 35 cycles of 95 °C for 30 s, 60 °C for 60 s, and 72 °C for 30 s, and a final extension at 72°C for 7 min. PCR products were separated on a 1.5% agarose gel by electrophoresis at 180 V for 30 min. The amplified fragments were purified using the LaboPass™ Gel and PCR Clean-up Kit (Cosmo Genetech Co., Seoul, Republic of Korea) and sequenced with the ABI Prism BigDye (ThermoFisher Scientific Inc., Waltham, MA, USA) by Macrogen (Macrogen Inc., Seoul, Republic of Korea).

### 2.4. Sequence Editing and Alignment

The amplified sequences of the mitochondrial genes COX1, ND5, and CYTB were inspected using ChromasPro (version 2.1.9, Technelysium Pty Ltd., South Brisbane, Australia), and low-quality base calls at the 5′ and 3′ ends were trimmed using BioEdit (version 7.2, Ibis Biosciences Co., Carlsbad, CA, USA). Forward and reverse reads were assembled into consensus sequences. Consensus sequences were aligned using MEGA X (version 10.2.6, megasoftware.net) with the MUSCLE algorithm [29], applying default gap-opening and gap-extension penalties. After alignment, all sequence fragments were trimmed to equal lengths for phylogenetic and haplotype network analyses.

### 2.5. Phylogenetic Analysis

Phylogenetic trees based on the aligned sequences retrieved from GenBank and those generated in this study were constructed using the maximum likelihood method under the Tamura–Nei model with 1,000 bootstrap replicates implemented in MEGA X [30].

### 2.6. Genetic Variation and Haplotype Network Analysis

Sequence comparison was conducted using the Needleman–Wunsch algorithm and the Smith–Waterman algorithm [31,32]. Population nucleotide diversity indices such as number of haplotypes, haplotype diversity (Hd), nucleotide diversity per site (π), average number of nucleotide differences (k), and number of polymorphic sites (S) were calculated using DnaSP (ver. 6.12.03, University of Barcelona, Spain) [33]. The haplotypes of the COX1 gene of *Ae. albopictus* were classified based on the KDCA study as a reference [26]. The pairwise distance of the haplotypes of *Ae. albopictus* was calculated using the MEGA X program.

The PopArt 1.7 (ver. 1.7, University of Otago, New Zealand) was used to construct a median-joining haplotype network analysis based on the region or country of source of *Ae. albopictus* to estimate the relationship between haplotypes using nexus input files produced by DnaSP version 6.12.03 [34].

## 3. Results

### 3.1. Genetic Differentiation and Diversity

To investigate the genetic diversity of *Ae. albopictus* populations, sequences of three mitochondrial genes (COX1, ND5, and CYTB) were aligned with datasets retrieved from GenBank. Two COX1 sequences (694 bp and 653 bp) were obtained from Korean *Ae. albopictus* were trimmed and aligned, and combined produce 48 sequence fragments with a total length of 1,347 bp. Additionally, 46 ND5 trimmed sequences (406 bp) and 47 CYTB trimmed sequences (472 bp) were aligned with corresponding GenBank data.

#### 3.1.1. Genetic Diversity of *Ae. albopictus* Based on COX1

Forty-eight COX1 sequences (1347 bp) of Korean *Ae. albopictus* were aligned, and haplotypes and genetic variation indices were analyzed, including COX1 sequences previously reported in Korea (Table 1). Analysis of the Korean COX1 sequences revealed nine haplotypes, six of which (H39–H44) were newly identified in this study. The H1 haplotype was predominant, occurring in 38 of the 48 sequences (79.2%).

**Figure 1:**
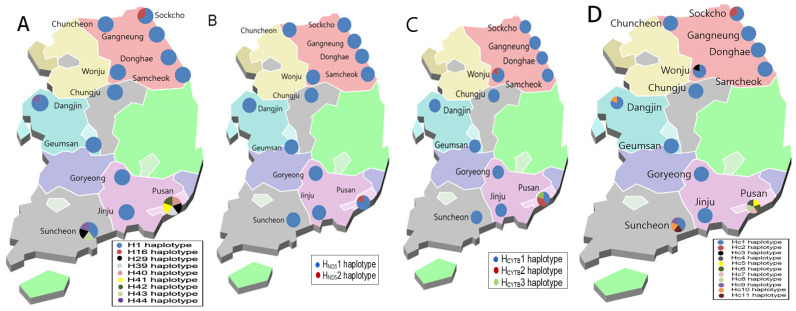
Geographic distribution of *Aedes albopictus* haplotypes in South Korea. A total of 4658 mosquitoes were collected from 13 sites across seven provinces, of which 542 *Ae. albopictus* were analyzed. Mitochondrial haplotypes were determined based on analyses of (**A**) COX1, (**B**) ND5, (**C**) CYTB, and (**D**) concatenated sequences. The relative proportions of haplotypes at each collection site are shown as pie charts. Colors corresponded to distinct haplotypes, highlighting regional variation in genetic diversity.

Regional haplotype distribution showed that most regions harbored a single dominant haplotype (H1) (Figure 1A). Notably, Busan exhibited the highest haplotype diversity, with five distinct haplotypes (Hd = 1.000), followed by Suncheon with four haplotypes (Hd = 0.900), and Sokcho with two (Hd = 0.667). Genetic diversity was detected nationwide, with particularly high variation observed in the southern coastal region, including Busan and Suncheon.

#### 3.1.2. Genetic Diversity of *Ae. albopictus* Based on ND5 and CYTB

Analysis of mitochondrial ND5 and CYTB sequences revealed low overall genetic diversity in Korean *Ae. albopictus*, with most variation concentrated in Busan and, to a lesser extent, Wonju (Table 2 and Table 3).

Across 46 ND5 sequences, only two haplotypes were identified, with H_ND5_1 overwhelmingly dominant (97.8%) and H_ND5_2 detected only in Busan. This indicates that ND5 variation is extremely limited and confined to specific regions. This contrasts with COX1, which showed broader variability across regions.

Similarly, CYTB analysis of 47 sequences revealed only 3 haplotypes, with H_CYTB_1 accounting for 93.6% of all samples. Additional haplotypes were observed only in Busan and Wonju, particularly Wonju, where COX1 showed no variation, but CYTB revealed distinct diversity. Although ND5 did not provide additional genetic diversity beyond that detected by COX1, CYTB exhibited higher diversity than COX1 in a certain region, suggesting that a multi-locus approach incorporating COX1 may be advantageous for assessing genetic diversity.

Overall, the ND5 and CYTB analyses indicated that the Korean *Ae. albopictus* population showed limited nationwide diversity, with genetic variation exclusively restricted to a specific region—especially Busan.

#### 3.1.3. Genetic Diversity of *Ae. albopictus* Based on Concatenated Sequences of COX1, ND5, and CYTB

To access the genetic diversity of the Korean *Ae. albopictus*, mitochondrial sequences of COX1, ND5, and CYTB were concatenated and analyzed. A total of 46 concatenated sequences were obtained, and 11 haplotypes (Hc1—Hc11) were identified (Table 4). The Hc1 haplotype was predominant and widely distributed nationwide. Moderate genetic diversity was observed in some regions, such as Sokcho and Wonju, where two haplotypes were detected. The highest diversity (Hd = 0.571) was observed in the southern coastal region. Busan exhibited the greatest haplotype richness, with five distinct haplotypes (Hc4—Hc8), all of which were unique to this region. Similarly, Suncheon showed relatively high genetic diversity, harboring four haplotypes (Hc1, Hc9, Hc10, and Hc11).

Overall, the concatenated mitochondrial datasets represent a key methodological advancement, providing greater discriminatory power than COX1-only analyses and enabling improved resolution of regional genetic variation and rare haplotypes.

### 3.2. Pairwise Genetic Distance of Ae. albopictus

#### 3.2.1. Pairwise Genetic Distance of *Ae. albopictus* Based on the COX1 Gene

Pairwise genetic distances between Korean *Ae. albopictus* COX1 haplotypes and a foreign haplotype from a dataset containing 28 sequences were analyzed (Table S3). Several Italian haplotypes were identical to the Korean H41 haplotype and were grouped accordingly. Within Korea, COX1 genetic distances were low (0.0000–0.0037), with the greatest divergence occurring between H40 and H42–H44, and between H16 and H40—both H40 and H42 being detected in Busan. The dominant haplotype H1 showed minimal divergence from most Korean haplotypes (0.0000–0.0022).

Between the regions, pairwise distances ranged from 0.0007 to 0.0098, with the highest values observed between Philippine haplotypes 1 and 4 and Taiwan. Relative to Korean haplotypes, H16, H42, and H43 showed the greatest divergence (0.0083) from Philippine haplotypes, followed by Taiwan (0.0060) and Thailand (0.0052). In contrast, genetic distances with neighboring populations from China (0.0007–0.0022) and Japan (0.0015–0.0037) were comparatively low.

#### 3.2.2. Pairwise Genetic Distance of *Ae. albopictus* Based on the ND5 and CYTB Gene

Pairwise genetic distances were analyzed between Korean *Ae. albopictus* ND5 haplotypes and foreign haplotypes from a dataset containing 38 sequences obtained from global regions. All foreign sequences matched the predominant Korean haplotype H_ND5_1 (Table S4). ND5 genetic distances ranged from 0.0025 to 0.2018, with the highest divergence (0.2018) observed between Taiwan (NC006817.1) and both H_ND5_2 and the Brazilian haplotype, indicating a unique Taiwanese lineage. All other pairwise distances, including that between H_ND5_1 and H_ND5_2 (0.0025), were very low.

For CYTB, comparisons with foreign haplotypes containing 16 sequences from Asia, Europe, Africa, and the Americas showed that many international sequences were identical to the dominant Korean haplotype H_CYTB_1 (Table S5). CYTB genetic distances ranged from 0.0000 to 0.0275, with the highest values observed between the Philippine haplotype 4 and Hawaii (0.0275), and between Korean H_CYTB_3 and Cambodia (0.0255). Within Korea, CYTB distances were low (0.0002–0.0125), indicating limited intraspecific variation.

#### 3.2.3. Pairwise Genetic Distance of *Ae. albopictus* Based on Concatenated Sequences of COX1, ND5, and CYTB

Pairwise genetic distances between haplotypes within Korean *Ae. albopictus* population, calculated by concatenating COX1, ND5, and CYTB sequences, were low, ranging from 0.0005 to 0.0032 (Table S6). The genetic distances between Korean haplotypes and those from other geographical regions were quite high. The highest genetic distance (0.0293–0.0312) was observed with the Taiwanese haplotype, while Southeast Asian populations, including the Philippines and Thailand, showed intermediate genetic distance.

Overall, pairwise distance analysis of concatenated mitochondrial sequence supports the usefulness of multi-locus mitochondrial data for resolving fine-scale phylogeographic relationships in the Asian tiger mosquito.

### 3.3. Haplotype Network Analysis

The haplotype network of Korean *Ae. albopictus* COX1, ND5, and CYTB sequences were analyzed using the TCS method, together with datasets obtained from GenBank. A total of 48 COX1, 46 ND5, and 47 CYTB sequences from Korean *Ae. albopictus* were included, along with 28, 39, and 16 foreign sequences for the respective genes.

#### 3.3.1. Haplotype Network Analysis of *Ae. albopictus* Based on COX1

**Figure 2:**
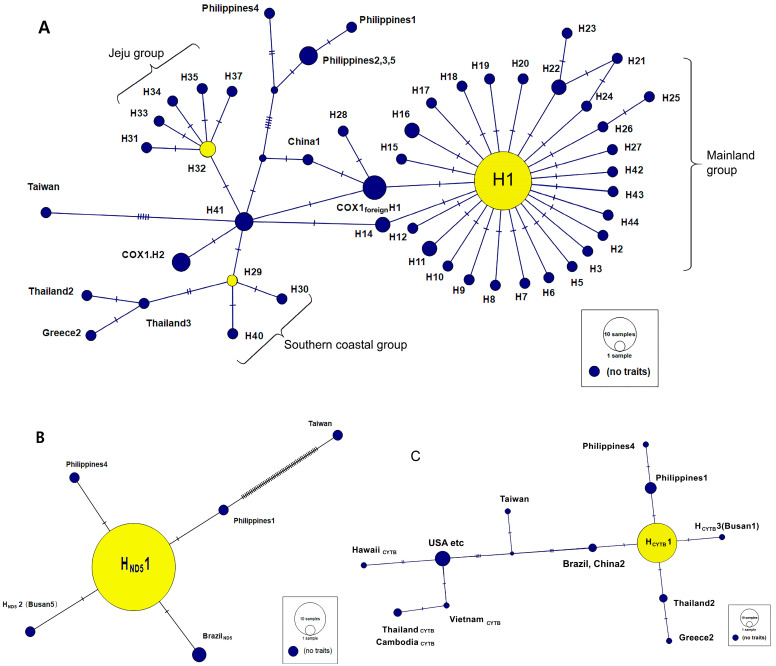
Haplotype network of Aedes albopictus COX1 (**A**), ND5 (**B**), and CYTB (**C**) sequences from Korea and datasets obtained from other regions, which include haplotypes from the Korean Disease Control and Prevention Agency (KDCA) and GenBank. Core haplotypes are highlighted in yellow.

The haplotype network of *Ae. albopictus* was constructed using 1,347 bp COX1 sequences generated by trimming and merging two amplicons, together with previously reported domestic and international haplotypes, including those from Jeju Island (Figure 2A). The haplotype network revealed three distinct groups—mainland, southern coastal, and Jeju group. The mainland group was centered on the core H1 haplotype, surrounded by multiple satellite haplotypes (H2–H27, H39, and H42–H44), and was only one base pair from a foreign H1 haplotype containing sequences from Europe, China, and Brazil. The southern coastal group was organized around H29, with H30 and H40 branching from it, and connected by one base pair to a Thailand haplotype. The Jeju group formed an isolated cluster centered on H32 and H36. The H41 haplotype (Busan 4) served as a key connector, linking the southern coastal (H29), Jeju (H32, H36), and mainland (via H14) groups, and further connecting to haplotypes from China, Japan, the Philippines, Portugal, and Taiwan. The bridge haplotype H41, detected in Busan—a major international port—strongly supports a port-mediated introduction and dispersal hypothesis, indicating that marine trade and human-mediated transport have played a significant role in shaping the genetic structure of *Ae. albopictus* populations in Korea.

#### 3.3.2. Haplotype Network Analysis of *Ae. albopictus* Based on ND5 and CYTB

A haplotype network of 87 ND5 sequences (397 bp) showed H_ND5_1 as the core haplotype, with the Korean H_ND5_2 and foreign haplotypes from the Philippines and Brazil differing by only one base pair. In contrast, the Taiwan_ND5_ haplotype was highly divergent, separated from Philippine 1 by 48 base pairs (Figure 2B).

For CYTB, analysis of 47 Korean and 86 foreign sequences (Figure 2C) identified H_CYTB_1 as the central haplotype. H_CYTB_2 and H_CYTB_3 differed from H_CYTB_1 by one base pair, and H_CYTB_3 showed no direct connection to foreign haplotypes. H_CYTB_1 clustered with haplotypes from the Philippines, Thailand, and China, whereas Taiwan, Brazil, and the “USA–etc.” cluster (Brazil, France, Lebanon, USA) were positioned at much greater genetic distances. Southeast Asian haplotypes (Vietnam, Thailand, Cambodia) also grouped with this distant cluster, remaining well separated from Korean haplotypes.

#### 3.3.3. Haplotype Network Analysis of *Ae. albopictus* Based on Concatenated Sequences of COX1, ND5, and CYTB

Haplotype network analysis based on concatenated sequences of *Ae. albopictus* identified two major groups: a mainland group and a southern coastal group (Figure S1). The mainland group was dominated by a central haplotype (Hc1) with several closely related satellite haplotypes (Hc2–Hc4 and Hc8–Hc10). The southern coastal group consisted mainly of haplotypes from Busan and southern Korea (Hc5, Hc6, and Hc11).

Haplotype Hc7 (Busan4, H41) linked the southern coastal group to Southeast Asian haplotypes from Thailand and the Philippines and was also connected to haplotypes from China, Japan, Italy, Portugal, Greece, and the United States. Overall, the concatenated network showed a pattern similar to that based on COX1 alone.

### 3.4. Phylogenetic and Genetic Distance

**Figure 3:**
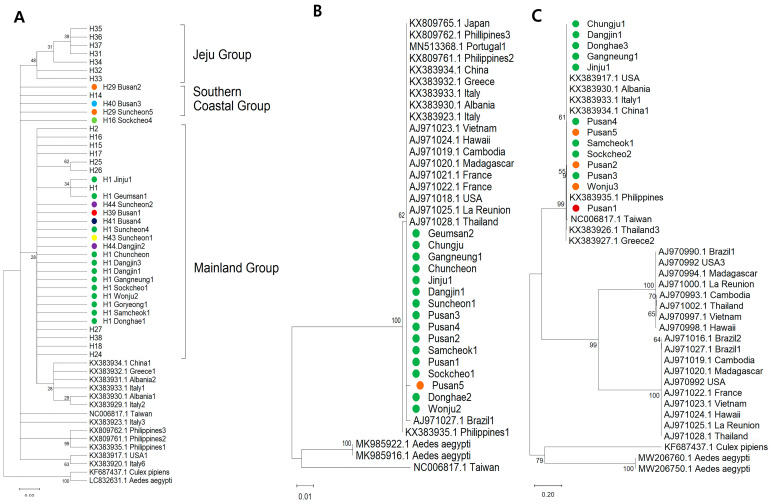
Phylogenetic trees of *Aedes albopictus* based on mitochondrial COX1 (**A**), ND5 (**B**), and CYTB (**C**) gene sequences. Phylogenetic analyses were conducted using the maximum-likelihood (ML) method based on the sequences obtained in this study and the dataset retrieved from GenBank. H_ND5_1 and H_CYTB_1 are shown in green, H_ND5_2 and H_CYTB_2 in orange, and H_CYTB_3 in red. H_ND5_1 and H_ND5_2 represent haplotypes based on the ND5 sequence of *Ae. albopictus*, whereas H_CYTB_1, H_CYTB_2, and H_CYTB_3 represent haplotypes based on the CYTB sequence of *Ae. albopictus*. The GenBank accession numbers for the *Ae. albopictus* COX1 sequences used in this study are provided in Table A1, while those for the ND5 and CYTB sequences are listed in Table A2.

To examine the phylogenetic relationships of *Ae. albopictus* populations, phylogenetic trees were constructed using the maximum likelihood method. Sequences of three mitochondrial genes—COX1, ND5, and CYTB—from *Aedes albopictus* were aligned and analyzed alongside datasets obtained from GenBank. A total of 48 COX1 sequences, 46 ND5 sequences, and 47 CYTB sequences of *Ae. albopictus* from Korea were aligned with corresponding datasets retrieved from GenBank. (Figure 3).

#### 3.4.1. Phylogenetic Analysis of *Ae. albopictus* Based on COX1

Phylogenetic analysis of COX1 sequences revealed three distinct clades of *Ae. albopictus* in Korea: the mainland, Jeju, and southern coastal groups (Figure 3A). The mainland group was distinct from those in China, Portugal, Brazil, Greece, and Albania. The Jeju group formed a clearly isolated cluster. The southern coastal group clustered near Thailand and Greece, but remained genetically distinct, showing no close relationship to geographically proximate Japan. The Philippines formed an independent and distinct cluster, with high support (bootstrap = 95).

#### 3.4.2. Phylogenetic Analysis of *Ae. albopictus* Based on ND5 and CYTB

A phylogenetic tree of ND5 sequences showed that the predominant Korean haplotype (H_ND5_1) and H_ND5_2 from Busan clustered closely with Philippine haplotypes 1 and 4 and the BrazilianND5 haplotype, whereas Taiwan sequences were more distantly related—consistent with the ND5 haplotype network (Figure 3B).

For CYTB, two major clades were identified: one comprising sequences from Korea, along with international sequences from the Philippines, Thailand, China, Brazil, and Greece, Italy, Albania, and the USA (Figure 3C). This broad clustering indicates that the dominant Korean CYTB haplotypes are part of a widely distributed global mitochondrial lineage. The second major clade branched separately with high bootstrap support, indicating an evolutionarily distinct mitochondrial lineage.

#### 3.4.3. Phylogenetic Analysis of *Ae. albopictus* Based on Concatenated Sequences of COX1, ND5, and CYTB

A phylogenetic tree based on concatenated sequences is divided into two major groups: the mainland group and the southern coastal group (Figure S2). The mainland population formed a relatively dense cluster consisting of haplotypes Hc1—Hc4 and Hc8—Hc10. In contrast, the southern coastal population included haplotypes Hc5, Hc6, and Hc11, which clustered with Southeast Asian and European haplotypes. Notably, Hc7 (Busan 4) was closely associated with overseas haplotypes, suggesting potential linkages between Korean and foreign populations. Overall, the phylogenetic patterns were consistent with the haplotype network analysis.

## 4. Discussion

In this study, we analyzed the phylogenetic diversity of the Asian tiger mosquito, *Ae. albopictus* in Korea using mitochondrial COX1, ND5, and CYTB markers, including analyses based on concatenated sequences. Our findings reveal important insights into the introduction pathways and regional dynamics of this globally invasive vector.

The COX1 marker of *Ae. albopictus* in Korea revealed various haplotypes, indicating that Korean *Ae. albopictus* population is genetically diverse rather than uniform [25,35]. COX1 analysis showed that southern coastal regions—particularly Busan and Suncheon—harbor the greatest haplotype diversity. This elevated diversity is a classic indicator of repeated introductions or ongoing gene flow from external sources [11]. Similar patterns have been observed in international port cities worldwide, where *Aedes* populations continually receive new lineages through trade and transportation [14]. Thus, the high diversity in Korea’s southern coastal regions likely reflects strong connectivity with foreign populations via maritime traffic [10,14]. In contrast, Jeju Island showed a distinct and isolated genetic cluster. Its differentiation mirrors patterns reported from geographically separated regions such as Malaysia, where local mosquito populations maintain unique genetic signatures despite potential dispersal [16]. This reinforces the idea that islands and ecologically isolated areas preserve region-specific lineages and may act as reservoirs of local diversity. ND5 and CYTB markers showed very limited diversity across Korea, possibly reflecting regional differences. Moreover, although these markers may be suitable for broad-scale diversity analyses, they appear less appropriate for fine-scale diversity assessments. Nonetheless, variation in CYTB sequences—similar to COX1—clearly characterized local diversification in the southern coastal region, and independent diversification was also found in the Wonju area. Analysis based on concatenated COX1, ND5, and CYTB sequences detected a larger number of haplotypes and higher haplotype diversity than analyses relying on COX1 alone, indicating improved genetic resolution. This suggests that integrating multiple mitochondrial loci captures a broader spectrum of intraspecific variation that may be overlooked by single-marker approaches. Importantly, the concatenated analysis enabled a nationwide characterization of genetic variation in *Ae. albopictus* populations across Korea (Figure 1D), providing a more comprehensive view of population connectivity and regional differentiation.

A key finding of this study is the identification of the H41 haplotype from Busan, which acts as a genetic bridge connecting all three domestic population groups. H41 also shows close relationships with lineages from China, Japan, and parts of Southeast Asia. The presence of such a “bridge haplotype” is biologically meaningful: it suggests that certain lineages may repeatedly enter Korea via major ports, subsequently dispersing domestically and linking otherwise distinct populations. This finding highlights Busan’s pivotal role as a national gateway for mosquito dispersal. Similar patterns have been documented in Europe, where highways, ports, and trade routes drive the rapid redistribution of Aedes mosquitoes across distant regions [11,13,14]. Phylogenetic analyses validated the three domestic groups and confirmed the intermediate, connector-like role of the Busan H41 haplotype among both Korean and foreign lineages. Together, these results reinforce the hypothesis that southern coastal Korea is experiencing continuous mosquito introductions, potentially influenced by increasing global trade, warming climates, and expanding mosquito habitat suitability [13].

Importantly, the identification of Busan as a genetic gateway has direct implications for vector surveillance strategies. Enhanced and sustained monitoring at international ports, cargo terminals, and surrounding urban areas should be prioritized to detect newly introduced lineages at an early stage. In contrast, the genetic distinctness of Jeju Island suggests limited gene flow with mainland populations, indicating that the island’s population may follow independent evolutionary and epidemiological trajectories. Therefore, Jeju Island should be monitored through separate, region-specific surveillance programs.

The broader implications of these findings are substantial. First, the strong genetic structuring in Korea suggests that *Ae. albopictus* populations are shaped by both natural geographic constraints and human-mediated movement. Second, the presence of genetically diverse lineages in port cities highlights the ongoing risk of introducing foreign vector populations—including those potentially carrying exotic arboviruses. Finally, the observed patterns align with global invasion models predicting the continued expansion of *Ae. albopictus* into new regions under climate change scenarios.

Collectively, our results emphasize the need for strengthened mosquito surveillance in coastal regions, targeted monitoring of introduction pathways, and integrated vector management strategies that account for both domestic population structure and international connectivity.

While mitochondrial COX1 remains a useful marker for species identification and broad phylogeographic comparisons in *Aedes* mosquitoes, its limited variability restricts its ability to resolve fine-scale population structure. In the present study, analyses based on concatenated COX1, ND5, and CYTB sequences partially overcome this limitation by detecting a greater number of haplotypes and higher haplotype diversity than COX1 alone. The concatenated approach enabled a more comprehensive assessment of nationwide genetic variation.

Nevertheless, mitochondrial markers represent a single, maternally inherited genome and therefore provide limited insight into complex demographic processes. In contrast, genome-wide SNPs, derived from thousands of nuclear loci, provide substantially higher resolution. Therefore, future detailed analyses of gene flow, population history, and microgeographic differentiation will benefit from the application of genome-wide SNP approaches [36].

## 5. Conclusions

In conclusion, this study highlights the complex phylogeographic structure and genetic diversity of *Ae. albopictus* in Korea. Analyses of COX1, ND5, and CYTB sequences revealed that southern coastal regions, particularly Busan, harbor the highest genetic diversity, likely reflecting repeated introductions and gene flow from foreign populations. Jeju Island populations formed a distinct cluster, emphasizing the influence of geographic isolation on local genetic structure. The identification of a “bridge haplotype” in Busan underscores the role of major ports in connecting domestic and international mosquito populations, facilitating both regional dispersal and potential long-distance introductions. These findings have direct implications for vector surveillance. As a genetic gateway, Busan and other major port cities should be prioritized for port-based inspections, early detection of introduced lineages, and targeted control. In contrast, the genetic distinctness of Jeju Island warrants separate, region-specific monitoring to track local population dynamics and prevent external introductions. Overall, southern coastal regions emerge as key entry points for *Ae. albopictus*, underscoring the need for enhanced surveillance, targeted vector management to mitigate the public health risks posed by this invasive mosquito species.

## Figures and Tables

**Table 1:** Haplotype and genetic diversity indices of *Aedes albopictus* populations based on COX1 sequences in Korea.

Province	Locality	N	H	Haplotypes	Hd	π	κ	S
Gangwon	Samcheok	5	1	H1	0.2047	0.0004	0.211	1
	Donghae	6	1	H1				
	Sockcheo	3	2	H1, H16				
	Gangneung	1	1	H1				
	Wonju	6	1	H1				
	Chuncheon	1	1	H1				
Chungbuk	Chungju	2	1	H1	0.333	0.0003	0.333	1
Chungnam	Dangjin	5	2	H1, H44				
	Geumsan	5	1	H1				
Jeonam	Suncheon	5	4	H1, H29, H43, H44	0.363	0.0003	0.468	8
Gyeongbuk	Goryeong	1	1	H1				
Gyeongnam	Jinju	3	1	H1				
Busan Metropolitan		5	5	H29, H39, H40, H41, H42				
Total		48	9	H1, H16, H29, H39~H44	0.341	0.0005	0.680	8

COX1, cytochrome *c* oxidase subunit 1; N, number of sequences; H, haplotype diversity; π, nucleotide diversity; κ, Average number of nucleotide differences; S, number of polymorphic sites. Hd, π, κ, and S were grouped by region—northeastern, central, and southern—and analyzed accordingly.

**Table 2:** Haplotype and genetic diversity indices of *Aedes albopictus* populations based on ND5 sequences in Korea.

Province	Locality	N	H	Haplotypes	Hd	π	κ	S
Gangwon	Samcheok	5	1	H_ND5_1	–	–	–	0
	Donghae	5	1	H_ND5_1				
	Sockcheo	3	1	H_ND5_1				
	Gangneung	1	1	H_ND5_1				
	Wonju	5	1	H_ND5_1				
	Chuncheon	1	1	H_ND5_1				
Chungbuk	Chungju	2	1	H_ND5_1	–	–	–	0
Chungnam	Dangjin	5	1	H_ND5_1				
	Geumsan	5	1	H_ND5_1				
Jeonam	Suncheon	5	1	H_ND5_1	0.111	0.291	0.111	1
Gyeongbuk	Goryeong	1	1	H_ND5_1				
Gyeongnam	Jinju	3	1	H_ND5_1				
Busan Metropolitan		5	2	H_ND5_1, H_ND5_2				
Total		46	2	H_ND5_1, H_ND5_2	0.043	0.0001	0.043	1

ND5, NADH dehydrogenase 5; N, number of sequences; H, haplotype diversity; π, nucleotide diversity; κ, Average number of nucleotide differences; S, number of polymorphic sites. Hd, π, κ, and S were grouped by region—northeastern, central, and southern—and analyzed accordingly.

**Table 3:** Haplotype and genetic diversity indices of *Aedes albopictus* populations based on CYTB sequences in Korea.

Province	Locality	N	H	Haplotypes	Hd	π	κ	S
Gangwon	Samcheok	6	1	H_CYTB_1	0.182	0.0009	0.341	0
	Donghae	6	1	H_CYTB_1				
	Sockcheo	3	1	H_CYTB_1				
	Gangneung	1	1	H_CYTB_1				
	Wonju	5	2	H_CYTB_1, H_CYTB_2				
	Chuncheon	1	1	H_CYTB_1				
Chungbuk	Chungju	2	1	H_CYTB_1	–	–	–	0
Chungnam	Dangjin	5	1	H_CYTB_1				
	Geumsan	5	1	H_CYTB_1				
Jeonam	Suncheon	5	1	H_CYTB_1	0.222	0.0010	0.222	2
Gyeongbuk	Goryeong	1	1	H_CYTB_1				
Gyeongnam	Jinju	3	1	H_CYTB_1				
Busan Metropolitan		5	3	H_CYTB_1, H_CYTB_2, H_CYTB_3				
Total		47	3	H_CYTB_1, H_CYTB_2, H_CYTB_3	0.0162	0.0004	0.165	2

CYTB; cytochrome *b*, N; number of sequences, H; haplotype diversity, π; nucleotide diversity, κ; Average number of nucleotide differences, S; number of polymorphic sites. Hd, π, κ, and S were grouped by region—northeastern, central, and southern—and analyzed accordingly.

**Table 4:** Haplotype and genetic diversity indices of *Aedes albopictus* populations based on concatenated sequences of COX1, ND5, and CYTB in Korea.

Province	Locality	N	H	Haplotypes	Hd	π	κ	S
Gangwon	Samcheok	5	1	Hc1	0.348	0.0002	0.371	3
	Donghae	5	1	Hc1				
	Sockcheo	3	2	Hc1, Hc2				
	Gangneung	1	1	Hc1				
	Wonju	5	2	Hc1, Hc3				
	Chuncheon	1	1	Hc1				
Chungbuk	Chungju	2	1	Hc1	0.167	0.0001	0.200	2
Chungnam	Dangjin	5	2	Hc1, Hc10				
	Geumsan	5	1	Hc1				
Jeonam	Suncheon	5	4	Hc1, Hc9, Hc10, Hc11	0.571	0.0004	0.876	9
Gyeongbuk	Goryeong	1	1	Hc1				
Gyeongnam	Jinju	3	1	Hc1				
Busan Metropolitan		5	5	Hc4, Hc5, Hc6, Hc7, Hc8				
Total		46	11	Hc1, Hc2, Hc3, Hc4, Hc5, Hc6, Hc7, Hc8, Hc9, Hc10, Hc11	0.424	0.0004	0.920	11

COX1, cytochrome *c* oxidase subunit 1; ND5, NADH dehydrogenase 5; CYTB, cytochrome *b*; N, number of sequences; H, haplotype diversity; π, nucleotide diversity; κ, Average number of nucleotide differences; S, number of polymorphic sites. Hd, π, κ, and S were grouped by region—northeastern, central, and southern—and analyzed accordingly.

## Data Availability

The original data used for the analyses can be obtained from the authors after approval by the responsible initiations in Korea.

## Supplementary Materials

The following supporting information can be downloaded at: https://www.mdpi.com/article/10.3390/insects17010082/s1, Table S1. Collection sites and collection of *Aedes albopictus* in Korea; Table S2. Primer sequences used for amplification of mitochondrial genes in *Aedes albopictus*; Table S3. Pairwise genetic distances of the haplotype of *Aedes albopictus* populations based on COX1 sequences in Korea and other regions; Table S4. Pairwise genetic distances of the haplotype of Aedes albopictus populations based on ND5 sequences in Korea and other regions; Table S5. Pairwise genetic distances of the haplotype of *Aedes albopictus* populations based on CYTB sequences in Korea and other regions; Table S6. Pairwise genetic distances of the haplotype of *Aedes albopictus* populations based on the concatenated sequence of COX1, ND5, and CYTB in Korea and other regions. Figure S1. Haplotype network of *Aedes albopictus* based on concatenated sequences of COX1, ND5, and CYTB from Korea and data set obtained from other regions. Core haplotypes are highlighted in yellow; Figure S2. Phylogenetic trees of *Aedes albopictus* based on concatenated sequences of COX1, ND5, and CYTB. Phylogenetic analyses were conducted using the maximum-likelihood (ML) method based on the sequences obtained in this study and data set retrieved from GenBank.

## Appendix A

**Table A1:** GenBank accession numbers of the COX1 sequences of *Ae. albopictus* in this study.

Locality Name	Gene	GenBank Accession Number	
Donghae_1	COX1	PX529948	PX518777
Donghae_2	COX1	PX529949	PX518778
Donghae_3	COX1	PX529950	PX518779
Donghae_5	COX1	PX529951	PX518780
Donghae_6	COX1	PX529952	PX518781
Donghae_7	COX1	PX529953	PX518782
Sockcheo_1	COX1	PX529954	PX518783
Sockcheo_2	COX1	PX529955	PX518784
Sockcheo_3	COX1	PX529956	PX518785
Sockcheo_4	COX1	PX529957	PX518786
Gangneung_1	COX1	PX529967	PX518797
Chuncheon	COX1	PX529976	PX518806
Samcheok_1	COX1	PX529958	PX518787
Samcheok_2	COX1	PX529959	PX518788
Samcheok_3	COX1	PX529960	PX518789
Samcheok_4	COX1	PX529961	PX518790
Wonju_2	COX1	PX529962	PX518792
Wonju_3	COX1	PX529963	PX518793
Wonju_4	COX1	PX529964	PX518794
Wonju_5	COX1	PX529965	PX518795
Wonju_6	COX1	PX529966	PX518796
Dangjin_1	COX1	PX529971	PX518801
Dangjin_2	COX1	PX529972	PX518802
Dangjin_3	COX1	PX529973	PX518803
Dangjin_4	COX1	PX529974	PX518804
Dangjin_5	COX1	PX529975	PX518805
Geumsan_1	COX1		PX518818
Geumsan_2	COX1		PX518819
Geumsan_3	COX1		PX518820
Geumsan_4	COX1		PX518821
Geumsan_5	COX1		PX518822
Suncheon_1	COX1		PX518807
Suncheon_2	COX1		PX518808
Suncheon_4	COX1		PX518809
Suncheon_5	COX1		PX518810
Goryeong_1	COX1		PX518798
Goryeong_2	COX1		PX518799
Goryeong_3	COX1		PX518800
Jinju_1	COX1		PX518815
Jinju_2	COX1		PX518816
Jinju_3	COX1		PX518817
Busan_1	COX1		PX518811
Busan_2	COX1		PX518812
Busan_3	COX1		PX518813
Busan_4	COX1		PX518814

**Table A2:** GenBank accession numbers of the ND5 and CYTB sequences of *Ae. albopictus* in this study.

Locality Name	GenBank Accession Number	
	ND5	CYTB
Donghae_2	PX511435	PX511394
Donghae_3	PX511436	PX511395
Donghae_5	PX511437	PX511397
Donghae_6	PX511438	PX511398
Donghae_7	PX511439	
Sockcheo_2		PX511399
Sockcheo_3	PX511441	PX511400
Sockcheo_4	PX511442	
Gangneung_1	PX511443	PX511401
Gangneung_2		PX511444
Chuncheon	PX511450	PX511402
Samcheok_1	PX511431	PX511389
Samcheok_2	PX511432	PX511390
Samcheok_3	PX511433	PX511391
Samcheok_4	PX511434	PX511392
Samcheok_6		PX511393
Wonju_2	PX511445	PX511403
Wonju_3	PX511446	PX511404
Wonju_4	PX511447	PX511405
Wonju_5	PX511448	PX511406
Wonju_6	PX511449	PX511407
Chungju_1	PX511451	PX511408
Dangjin_1	PX511452	PX511409
Dangjin_2	PX511453	PX511410
Dangjin_3	PX511454	PX511411
Dangjin_4	PX511455	PX511412
Dangjin_5	PX511456	PX511413
Dangjin_7		PX511414
Geumsan_1	PX511457	PX511415
Geumsan_2	PX511458	PX511416
Geumsan_3	PX511459	PX511417
Geumsan_4	PX511460	PX511418
Geumsan_5	PX511461	
Suncheon_1	PX511462	PX511419
Suncheon_2	PX511463	PX511420
Suncheon_3	PX511464	PX511421
Suncheon_4	PX511465	
Suncheon_5	PX511466	
Goryeong_1		PX511422
Jinju_1	PX511467	PX511423
Jinju_2	PX511468	PX511424
Jinju_3	PX511469	PX511425
Busan_1	PX511470	PX511426
Busan_2	PX511471	PX511427
Busan_3	PX511472	PX511428
Busan_4	PX511473	PX511429
Busan_5	PX511474	PX511430
