# Supplementary material for: Phylogeographic Insights into Aedes albopictus in Korea: Integrating COX1, ND5, and CYTB Analyses
Source: Insects. 2026 Jan 10;17(1):82. doi: 10.3390/insects17010082 (PMC12842514; doi:10.3390/insects17010082)
Supplement: Supplementary file 1 [file insects-17-00082-s001.zip › insects-3953550-supplementary.pdf]

Table S1. Collection sites and collection of *Aedes albopictus* in Korea

Province	Locality	GPS (latitude, longitude)	No. of collected mosquitoes	No. of *Ae. albopictus*	No. of *Ae. albopictus* used for analysis
Gangwon	Chuncheon	37.87946836, 127.7696376	21	1	1
	Donghae	37.51992280, 129.1182695	865	168	7
	Gangneung	37.69058631, 128.912241	21	1	1
	Samcheok	37.38143263, 129.0948147	498	94	6
	Sockcheo	38.20345919, 128.5713057	812	106	4
	Wonju	37.34248505, 127.9962678	2181	136	9
Chungbuk	Chungju	36.99867589, 127.9190799	17	2	2
Chungnam	Dangjin	36.93415869, 126.5960473	26	5	5
	Geumsan	36.14297704, 127.4861713	18	13	5
Jeonam	Suncheon	34.90467839, 127.5071745	93	7	5
Gyeongbuk	Goryeong	35.67863331, 128.1946102	2	1	1
Gyeongnam	Jinju	35.20236794, 128.1074913	11	3	3
Pusan Metropolitan		35.10371227, 128.9423860	93	5	5
Total			4,658	542	54

Table S2. Primer sequences used for amplification of mitochondrial genes of *Aedes albopictus*.

Target gene	Primer	Sequences (5′-3′)	Size (bp)	Reference
COX1	LCO1490	GGTCAACAAATCATAAAGATATTGG	710	[29]
	HCO2198	TAAACTTCAGGGTGACCAAAAAATCA		
	COX1-F	AAAAAGGAAACTTTTGGTACTTTAGGA	817	This study
	COX1-R	AAGAAAGAAGAGGTAATTCTGAATAGG		
ND5	F6500	TCCTTAGAATAAAATCCCGC	450	[30]
	R7398	GTTTCTGCTTTAGTTCATTCTTC		
CYTB	CYTB-F	GGCCCATTACGACCAATAAA	538	This study
	CYTB-R	TCAACAGCAAATCCTCCTCA		

Table S3. Pairwise genetic distances of haplotype of *Aedes albopictus* populations based on COX1 sequences in Korea and other regions.

Haplotypes	H1	H16	H29	H39	H40	H41	H42	H43	H44	Taiwan	Portugal1	Portu-	Japan	Philip-	Philip-	Philip-	China1	Thailand1	Greece1	Thailand2	Thailand3	Italy6,7,
H1	–																					
H16	0.0007	–																				
H39	0.0000	0.0007	–																			
H29	0.0022	0.0030	0.0022	–																		
H40	0.0030	0.0037	0.0030	0.0007	–																	
H41	0.0015	0.0022	0.0015	0.0007	0.0015	–																
H42	0.0007	0.0015	0.0007	0.0030	0.0037	0.0022	–															
H43	0.0007	0.0015	0.0007	0.0030	0.0037	0.0022	0.0015	–														
H44	0.0007	0.0015	0.0007	0.0030	0.0037	0.0022	0.0015	0.0015	–													
Taiwan	0.0052	0.0060	0.0052	0.0045	0.0052	0.0037	0.0060	0.0060	0.0060	–												
Portugal1	0.0037	0.0045	0.0037	0.0030	0.0037	0.0022	0.0045	0.0045	0.0045	0.0060	–											
Portugal2,†	0.0007	0.0015	0.0007	0.0015	0.0022	0.0007	0.0015	0.0015	0.0015	0.0045	0.0030	–										
Japan	0.0030	0.0037	0.0030	0.0022	0.0030	0.0015	0.0037	0.0037	0.0037	0.0052	0.0007	0.0022	–									
Philippines4	0.0075	0.0083	0.0075	0.0067	0.0075	0.0060	0.0083	0.0083	0.0067	0.0098	0.0083	0.0067	0.0075	–								
Philip-pines2,3,5	0.0067	0.0075	0.0067	0.0060	0.0067	0.0052	0.0075	0.0075	0.0060	0.0090	0.0075	0.0060	0.0067	0.0022	–							
Philippines1	0.0075	0.0083	0.0075	0.0067	0.0075	0.0060	0.0083	0.0083	0.0067	0.0098	0.0083	0.0067	0.0075	0.0030	0.0007	–						
China1	0.0015	0.0022	0.0015	0.0022	0.0030	0.0015	0.0022	0.0022	0.0022	0.0052	0.0037	0.0007	0.0030	0.0060	0.0052	0.0060	–					
Thailand1	0.0030	0.0037	0.0030	0.0007	0.0015	0.0015	0.0037	0.0037	0.0037	0.0052	0.0037	0.0022	0.0030	0.0075	0.0067	0.0075	0.0030	–				
Greece2	0.0045	0.0052	0.0045	0.0022	0.0030	0.0030	0.0052	0.0052	0.0052	0.0068	0.0052	0.0037	0.0045	0.0075	0.0067	0.0075	0.0030	0.0015	–			
Thailand2	0.0045	0.0052	0.0045	0.0022	0.0030	0.0030	0.0052	0.0052	0.0052	0.0068	0.0052	0.0037	0.0045	0.0075	0.0067	0.0075	0.0030	0.0015	0.0015	–		
Thailand3	0.0037	0.0045	0.0037	0.0015	0.0022	0.0022	0.0045	0.0045	0.0045	0.0060	0.0045	0.0030	0.0037	0.0067	0.0060	0.0067	0.0022	0.0007	0.0007	0.0007	–	
Italy6,7, USA1,2	0.0022	0.0030	0.0022	0.0015	0.0022	0.0007	0.0030	0.0030	0.0030	0.0045	0.0030	0.0015	0.0022	0.0067	0.0060	0.0067	0.0022	0.0022	0.0037	0.0037	0.0030	–

COX1: cytochrome c oxidase subunit 1

Portugal2,†: Portugal2, Brazil, Italy1,2, Greece1, Albania1,2, China2

COX1 haplotypes were obtained from GenBank: Taiwan (NC006817.1); Portugal1, 2 (MN513368.1, MN513355.1); Brazil (MN575475.1); Italy1,2,3,4,5,6,7,8 (KX383933.1, KX383929.1, KX383923.1, KX383922.1, KX383921.1, KX383920.1, KX383918.1, KX383916.1); Greece1, 2 (KX383932.1, KX383927.1); Albania1, 2 (KX383930.1, KX383931.1); China1, 2 (KX383934.1, KR068634.1); Japan (KX809765.1); Philippines1, 2, 3, 4, 5 (KX383935.1, KX809761.1, KX8009762.1, KX8009763.1, KX8009764.1); Thailand1, 2, 3 (KX382928.1, KX383925.1, KX383926.1); USA1, 2 (KX383917.1, KX383919.1).

Table S4. Pairwise genetic distances of haplotype of *Aedes albopictus* populations based on ND5 sequences in Korea and other regions.

Haplotypes	$H_{ND5}1$	$H_{ND5}2$	Taiwan	Philippines4	Philippines1	$Brazil_{ND5}$
$H_{ND5}1$	–					
$H_{ND5}2$	0.0025	–				
Taiwan	0.1958	0.2018	–			
Philippines4	0.0025	0.0051	0.1958	–		
Philippines1	0.0025	0.0051	0.1904	0.0051	–	
$Brazil_{ND5}$	0.0025	0.0051	0.2018	0.0051	0.0051	–

ND5: NADH dehydrogenase subunit 5

Reference ND5 haplotypes were obtained from GenBank: Taiwan (NC006817.1); Philippines1, 4 (KX383935.1, KX8009763.1); $Brazil_{ND5}$ (AJ971027.1, AJ971016.1)

Table S5. Pairwise genetic distances of haplotype of *Aedes albopictus* populations based on CYTB sequences in Korea and other regions.

Haplotypes	$H_{CYTB}1$	$H_{CYTB}2$	$H_{CYTB}3$	Taiwan	USA etc†	Philippines1,2,3,5	Philippines4	Greece2, Thailand2,3	$Thailand_{CYTB}$, Cambodia	Brazil, China2	Hawaii	Vietnam
$H_{CYTB}1$	–											
$H_{CYTB}2$	0.0000	–										
$H_{CYTB}3$	0.0020	0.0020	–									
Taiwan	0.0104	0.0104	0.0125	–								
USA etc†	0.0020	0.0020	0.0041	0.0082	–							
Philippines1,2,3,5	0.0020	0.0020	0.0041	0.0125	0.0041	–						
Philippines4	0.0041	0.0041	0.0061	0.0146	0.0062	0.0020	–					
Greece2, Thailand2,3	0.0000	0.0000	0.0020	0.0104	0.0020	0.0020	0.0041	–				
$Thailand_{CYTB}$, Cambodia	0.0233	0.0233	0.0255	0.0167	0.0211	0.0211	0.0233	0.0233	–			
Brazil, China2	0.0190	0.0190	0.0211	0.0124	0.0168	0.0211	0.0232	0.0190	0.0041	–		
Hawaii	0.0232	0.0232	0.0254	0.0166	0.0210	0.0254	0.0275	0.0232	0.0082	0.0041	–	
Vietnam	0.0211	0.0211	0.0233	0.0145	0.0189	0.0190	0.0211	0.0211	0.0020	0.0020	0.0061	–

CYTB: cytochrome *b*

Reference CYTB haplotypes were obtained from GenBank: Taiwan (NC006817.1), Brazil (MN575475.1), Greece2 (KX383927.1), China2 (KR068634.1), Philippines1, 2, 3, 4, 5 (KX383935.1, KX809761.1, KX8009762.1, KX8009763.1, KX8009764.1), Thailand2,3 (KX383925.1, KX383926.1), $Thailand_{CYTB}$ (AJ971002.1), Cambodia (AJ970993.1), Lebanon (JX971201.1), Hawaii (AJ970998.1), Vietnam (AJ970997.1).

USA etc†: USA (AJ970992), Brazil (AJ970990, AJ971001), Lebanon (JX971201), La Providence reunion (AJ971000), France (AJ970996), and Madagascar (AJ970994).

Table S6. Pairwise genetic distances of haplotype of *Aedes albopictus* populations based on concatenated sequence of COX1, ND5, and CYTB in Korea and other regions.

Haplotypes	Hc1	Hc2	Hc3	Hc4	Hc5	Hc6	Hc7	Hc8	Hc9	Hc10	Hc11	Taiwan	Portugal1	Portugal2,†	Brazil2, China2	Japan	Philippines2,3,5	Philippines4	Philippines1	China1	Greece2	Tailand3	Thailand2	Italy4,5	Italy6,7 USA1,2
Hc1	–																								
Hc2	0.0005	–																							
Hc3	0.0005	0.0009	–																						
Hc4	0.0005	0.0009	0.0009	–																					
Hc5	0.0018	0.0023	0.0014	0.0023	–																				
Hc6	0.0023	0.0027	0.0018	0.0027	0.0005	–																			
Hc7	0.0009	0.0014	0.0014	0.0014	0.0009	0.0014	–																		
Hc8	0.0009	0.0014	0.0014	0.0014	0.0027	0.0032	0.0018	–																	
Hc9	0.0005	0.0009	0.0007	0.0009	0.0023	0.0027	0.0014	0.0014	–																
Hc10	0.0005	0.0009	0.0009	0.0009	0.0023	0.0027	0.0014	0.0014	0.0009	–															
Hc11	0.0014	0.0018	0.0018	0.0018	0.0005	0.0009	0.0005	0.0023	0.0018	0.0018	–														
Taiwan	0.0303	0.0307	0.0307	0.0307	0.0303	0.0307	0.0293	0.0312	0.0307	0.0307	0.0298	–													
Portugal1	0.0023	0.0027	0.0027	0.0027	0.0023	0.0027	0.0014	0.0032	0.0027	0.0027	0.0018	0.0307	–												
Portugal2,†	0.0005	0.0009	0.0009	0.0009	0.0014	0.0018	0.0005	0.0014	0.0009	0.0009	0.0009	0.0298	0.0018	–											
Brazil2, China2	0.009	0.0014	0.0014	0.0014	0.0018	0.0023	0.0009	0.0018	0.0014	0.0014	0.0014	0.0293	0.0023	0.0005	–										
Japan	0.0018	0.0023	0.0023	0.0023	0.0018	0.0023	0.0009	0.0027	0.0023	0.0023	0.0014	0.0303	0.0005	0.0014	0.0018	–									
Philippines2,3,5	0.0045	0.0050	0.0050	0.0050	0.0045	0.0050	0.0036	0.0054	0.0050	0.0041	0.0041	0.0331	0.0050	0.0041	0.0045	0.0045	–								
Philippines4	0.0059	0.0063	0.0063	0.0063	0.0059	0.0063	0.0050	0.0068	0.0063	0.0054	0.0054	0.0341	0.0063	0.0054	0.0059	0.0059	0.0023	–							
Philippines1	0.0054	0.0059	0.0059	0.0059	0.0054	0.0059	0.0045	0.0063	0.0059	0.0050	0.0050	0.0331	0.0059	0.0050	0.0054	0.0054	0.0009	0.0032	–						
China1	0.0009	0.0014	0.0014	0.0014	0.0018	0.0023	0.0009	0.0018	0.0014	0.0014	0.0014	0.0303	0.0023	0.0005	0.0009	0.0018	0.0036	0.0050	0.0045	–					
Greece2	0.036	0.0041	0.0041	0.0041	0.0027	0.0032	0.0027	0.0045	0.0041	0.0041	0.0023	0.0034	0.0322	0.0032	0.0036	0.0036	0.0054	0.0068	0.0063	0.0027	–				
Thailand3	0.0027	0.0032	0.0032	0.0032	0.0018	0.0023	0.0018	0.0036	0.0032	0.0032	0.0014	0.0312	0.0032	0.0023	0.0027	0.0027	0.0045	0.0059	0.0054	0.0018	0.0009	–			
Thailand2	0.0032	0.0036	0.0036	0.0036	0.0023	0.0027	0.0023	0.0041	0.0036	0.0036	0.0018	0.0317	0.0036	0.0027	0.0032	0.0032	0.0050	0.0063	0.0059	0.0023	0.0014	0.0005	–		
Italy4,5	0.0014	0.0018	0.0018	0.0018	0.0014	0.0018	0.0005	0.0023	0.0018	0.0018	0.0009	0.0298	0.0018	0.0009	0.0014	0.0014	0.0041	0.0054	0.0050	0.0050	0.0014	0.0032	0.0023	0.0027	
Italy6,7, USA1,2	0.0018	0.0023	0.0023	0.0023	0.0018	0.0023	0.0009	0.0027	0.0023	0.0023	0.0014	0.0303	0.0023	0.0014	0.0018	0.0018	0.0045	0.0059	0.0054	0.0018	0.0036	0.0027	0.0032	0.0030	–

COX1: cytochrome *c* oxidase subunit 1; ND5, NADH dehydrogenase 5; CYTB, cytochrome *b*; Portugal2,†: Portugal2, Brazil, Italy1,2, Greece1, Albania1,2, China2

COX1 haplotypes were obtained from GenBank: Taiwan (NC006817.1); Portugal1, 2 (MN513368.1, MN513355.1); Brazil (MN575475.1); Italy1,2,3,4,5,6,7,8 (KX383933.1, KX383929.1, KX383923.1, KX383922.1, KX383921.1, KX383920.1, KX383918.1, KX383916.1); Greece1, 2 (KX383932.1, KX383927.1); Albania1, 2 (KX383930.1, KX383931.1); China1, 2 (KX383934.1, KR068634.1); Japan (KX809765.1); Philippines1, 2, 3, 4, 5 (KX383935.1, KX809761.1, KX8009762.1, KX8009763.1, KX8009764.1); Thailand1, 2, 3 (KX382928.1, KX383925.1, KX383926.1); USA1, 2 (KX383917.1, KX383919.1).

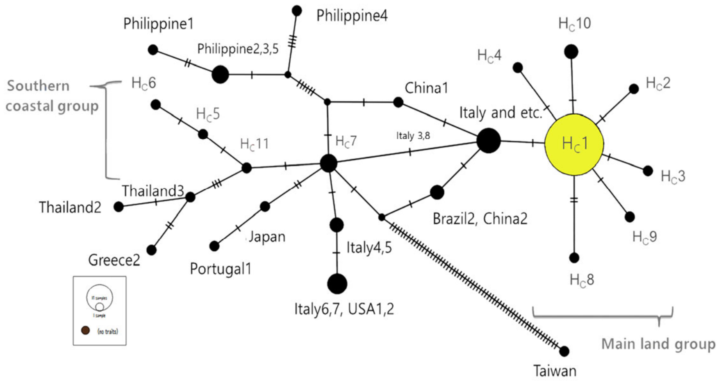

Figure S1. Haplotype network of *Aedes albopictus* based on concatenated sequences of COX1, ND5, and CYTB from Korea and data set obtained from other regions. Core haplotypes are highlighted in yellow.

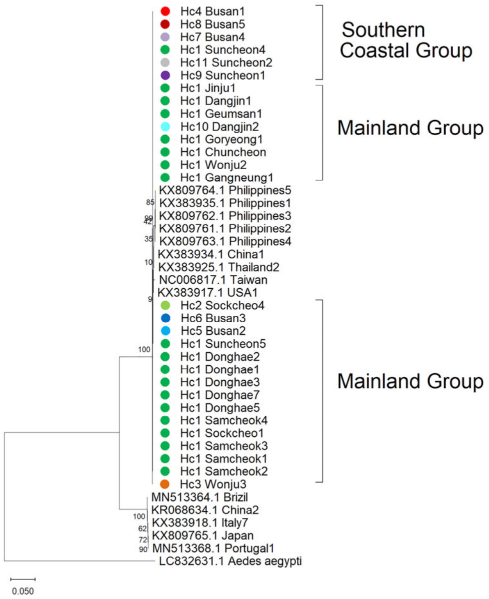

Figure S2. Phylogenetic trees of *Aedes* **albopictus** based on concatenated sequences of COX1, ND5, and CYTB. Phylogenetic analyses were conducted using the maximum-likelihood (ML) method based on the sequences obtained in this study and data set retrieved from GenBank.
